# The role of foundations: Rockefeller Foundation

**DOI:** 10.1186/s40985-016-0041-4

**Published:** 2016-12-12

**Authors:** David Rockefeller

**Affiliations:** Rockefeller Foundation, New York, NY USA

**Keywords:** Climate change, Public health, Planetary health, Foundations, Future health

## Abstract

The consequences of climate change and the impacts of human activity on the environment have made it clearer than ever before that we must evolve our current model of public health to better account for the inextricable link between human health and the natural systems on which it depends—creating a “public health 2.0” that builds on the innovations of the twentieth century to account for a world where humans have bypassed planetary boundaries to achieve well-being. First coined at the Rockefeller Foundation’s Centennial gathering in Beijing in 2013, “Planetary Health” will factor in future health and environmental harms over present-day gains, particularly those that disproportionately affect the poor and those in developing nations. To build this new field, foundations must address the challenge of information, increasing support for research to bridge knowledge gaps on the links between economic development, natural systems, and human health.

## Main text

Thank you Jose, Stefanie, and of course Sarah. And thanks to Antoine and his team for organizing this event during a very packed 2 weeks, to say the least.

By now, we have heard the compelling case, eloquently made, for evolving our current model of public health to better account for the inextricable link between human health and the natural ecosystems, the natural systems on which life depends.

This was the seminal idea that emerged from the Rockefeller Foundation’s Centennial gathering in Beijing in 2013. We met there at a critical time. The consequences of climate change were already making themselves apparent in vulnerable places around the world. The impacts of human activity were equally evident, nowhere more than in the exploitation and acidification of our most precious resource, our oceans. A pet resource of mine. Meanwhile, seven million deaths were being attributed to air pollution; new diseases were emerging; and patterns of known diseases were changing. And this was before Ebola confirmed to us growing anxieties about the dangers of disease transmission from animal to human and the quickness by which it spreads in a globalized world.

And so, it was no far-flung conclusion that we needed a new frame for public health in the twenty-first century—a “public health 2.0,” if you will.

Do not misunderstand me. As the great-grandson of the man whose foundation pioneered in many ways the modern field of public health, I am quite fond of the original version, Public Health 1.0. And I am proud, as we all are, of the progress it has helped us achieve, from wiping out diseases like hookworm and yellow fever. Humanity has never been in greater health. But we have also never been in greater peril. So we need a health framing that accounts for both, which is what planetary health offers.

But two big challenges are standing in our way.

The first, as has been said before, is one of the imagination. For the last 85 years, the world has relied on gross domestic product as the chief indicator of human progress. But we have failed to account for future health and environmental harms over present-day gains, with the disproportionate effect of those harms on the poor and those in developing nations. Foundations can lead the way in helping to establish new indicators that measure human progress by balancing economic development with advancing human health and protecting the environment.

Second is a challenge of research and information. There is still so much we do not know when it comes to understanding the social and environmental drivers of human health. Foundations must increase our support for the inter- and trans-disciplinary research necessary to bridge these gaps and recognize the links between economic development, natural systems, and human health. Which is why I am delighted today to announce the official launch of the Rockefeller Foundation funded Planetary Health Alliance—a network of researchers who will develop curricula to train the world’s first planetary health scientists, convene scholars around the world, and conduct advocacy work to ensure an enabling policy environment for planetary health. The alliance will have a base at Harvard University but will welcome minds and scholars from around the globe.

We are excited at the promise here. Planetary health offers an unprecedented opportunity for advocacy of global and national reforms, and foundations like ours and Wellcome will play a key role in launching a new approach to health for the next century. We look forward to working with everyone in this room and other rooms to make that a reality, and I look forward to discussing exactly how we might begin alongside the esteemed Sarah Molton.

Thank you very much.

